# Supplementary material for: Insights into Characteristic Volatiles in Wuyi Rock Teas with Different Cultivars by Chemometrics and Gas Chromatography Olfactometry/Mass Spectrometry
Source: Foods. 2022 Dec 19;11(24):4109. doi: 10.3390/foods11244109 (PMC9777755; doi:10.3390/foods11244109)
Supplement: Supplementary file 1 [file foods-11-04109-s001.zip › foods-2085319-supplementary.pdf]

Insights into Characteristic Volatiles in Wuyi Rock Teas with Different Cultivars by Chemometrics and Gas Chromatography Olfactometry/Mass Spectrometry

Yue Zhang[1], Suyoung Kang[1], Han Yan[1], Dongchao Xie[1], Qincao Chen[1], Haipeng Lv[1], Zhi Lin[1,*], Yin Zhu[2,*]

[1] Tea Research Institute, Chinese Academy of Agricultural Sciences, Hangzhou 310008, China
[2] Key Laboratory of Tea Processing Engineering of Zhejiang Province, Hangzhou 310008, China
* Correspondence: linzhi@caas.cn (Z.L.); zhuy_scu@tricaas.com (Y.Z.)

Supplementary Material

Table S1. The peak area of the identified aroma components in Wuyi rock teas (WRTs) with different cultivars.

No	Name	CAS	RI (ref) [a]	RI (cal) [b]	Peak areas			
					DHP	RG	SX	JF
Esters								
1	Methyl isovalerate	556-24-1	773	781	215662±100949	79147±49524	139846±82489	94680±49205
2	Methyl 2-methylbutyrate	868-57-5	774	782	114594±58865	47914±38918	74935±54065	44227±20262
3	Methyl hexanoate	106-70-7	925	924	4609501±1532654	1743876±393411	2545066±664129	2165534±938023
4	(*Z*)-Methyl 3-hexenoate	13894-62-7	933	933	1166262±330467	726980±131116	551960±212938	526991±120256
5	Methyl 2-furoate	611-13-2	979	976	12495614±2221408	6472564±1800302	8127904±4823523	6831981±2333429
6	Methyl heptanoate	106-73-0	1026	1025	595456±228371	279792±68259	375866±95197	390676±85557
7	Methyl benzoate	93-58-3	1094	1092	2430607±369735	2194414±301278	1605464±523199	1633592±317383
8	Methyl octanoate	111-11-5	1126	1124	820896±287863	449074±71056	531617±91286	652510±162856
9	Benzyl acetate	140-11-4	1162	1164	1418984±210207	1472806±274444	1059015±313191	1450675±326922
10	Methyl phenylacetate	101-41-7	1179	1178	3165717±586816	2266794±611576	1882388±839847	1245161±333102
11	Hexyl butyrate	2639-63-6	1192	1190	1038341±392821	939460±104800	575360±247305	1903387±830490
12	Methyl salicylate	119-36-8	1193	1194	28399079±5493348	24047830±3410996	19172166±5520229	17619983±2449918
13	Methyl nonanoate	1731-84-6	1227	1222	344387±92877	174976±16546	253244±55865	248840±67999
14	*cis*-3-Hexenyl-*α*-methylbutyrate	53398-85-9	1234	1234	383797±89700	241487±154249	324561±173468	179441±155869
15	Hexyl 2-methylbutyrate	10032-15-2	1236	1235	2443478±661736	2988161±447269	1508937±649329	1924754±629018
16	*trans*-2-Hexenyl isovalerate	68698-59-9	1245	1237	480320±117684	457957±129339	338011±156134	274559±122088
17	Phenethyl acetate	103-45-7	1245	1255	3434153±503415	5377172±900934	1869663±574270	3511332±844494
18	(*E*)-Methylgeranate	1189-09-9	1324	1321	442654±99825	403158±60003	295669±76212	448722±93536
19	*cis*-3-Hexenyl hexanoate	31501-11-8	1376	1379	16977914±3742162	23399825±3086880	9648998±3902470	22849855±7040705
20	Hexyl hexanoate	6378-65-0	1387	1384	7989405±1555974	9510381±401103	4546533±1632817	9727157±3532711
21	(*E*)-2-Hexenyl hexanoate	53398-86-0	1391	1387	6329479±1316822	7274963±1042215	4113090±1524604	7231242±2820050
22	Phenethyl butyrate	103-52-6	1444	1439	2304502±676382	5748276±895348	1235717±678674	6175686±2385852
23	Phenethyl 2-methylbutyrate	24817-51-4	1488	1486	2472061±662061	6484036±1354281	880256±329382	2077989±354399
24	*cis*-3-Hexenyl benzoate	25152-85-6	1580	1570	1669496±559557	5182144±1391668	1471074±946816	5316329±1893112
25	*cis*-3-Hexenyl octanoate	61444-41-5	1562	1576	337729±70362	498980±144129	234158±101018	763779±280295

26	Hexyl benzoate	6789-88-4	1580	1577	1416688±505382	3540801±753985	1129167±669315	3690262±1499336
27	(*E*)-2-Hexenyl benzoate	76841-70-8	1588	1584	887337±355796	2385386±445019	782997±496668	2083966±896539
28	Benzyl benzoate	120-51-4	1766	1763	24091±8052	56682±16314	39748±28540	51134±14410
29	Methyl hexadecanoate	112-39-0	1926	1921	815691±253343	1032454±303200	708561±294164	1146322±219210
	Alkenes							
1	Styrene	100-42-5	895	899	2393928±542041	1382993±322930	1671144±450588	1078584±341610
2	α-Pinene	2437-95-8	937	935	162842±52108	107867±36358	142726±62240	121746±44066
3	1-Propylcyclohexene	2539-75-5	949	944	1227308±611289	740604±309042	761754±222556	387684±127250
4	3,5,5-Trimethyl-2-hexene	26456-76-8	985	973	260950±39666	289305±53056	295936±60717	417520±191788
5	Myrcene	123-35-3	992	993	5608195±1200685	4915440±1458488	5028603±2676591	5374194±2517376
6	α-Phellandrene	99-83-2	1005	1007	299861±88826	314168±111574	275183±175009	357507±184905
7	δ-Carene	29050-33-7	1018	1017	1789975±509559	1618085±500342	1199475±559364	1299894±593136
8	Limonene	138-86-3	1030	1030	10562554±2281351	9777209±2076282	7181888±2302109	9396558±1781736
9	*cis*-β-Ocimene	3338-55-4	1038	1038	1748040±390145	1773477±586716	1887648±1282004	2045185±1146225
10	*trans*-β-Ocimene	3779-61-1	1049	1048	196901±47804	196098±80081	189613±111399	224964±123157
11	2,3,6-Trimethyl-1,5-heptadiene	33501-88-1	1055	1053	388800±88581	414558±78794	314916±99032	692625±341429
12	*m*-Cymenene	1124-20-5	1085	1089	2462281±400872	2045894±361766	1759386±443889	1687609±383340
13	(*4E,6Z*)-2,6-Dimethyl-2,4,6-octatriene	7216-56-0	1131	1128	1190154±280421	971571±204283	768368±251039	1203055±627618
14	*trans*-Alloocimene	3016-19-1	1144	1140	1367976±400486	1234092±240699	805555±233870	1405286±777923
15	(*E*)-4-(2-Butenyl)-1,2-dimethyl-benzene	54340-86-2	1311	1293	588547±155072	429573±160025	355931±263231	417478±182281
16	α-Cedrene	469-61-4	1418	1414	833148±193201	1323065±966066	797828±431021	744960±177414
17	(*E*)-β-Famesene	18794-84-8	1460	1455	1368102±365098	2728262±323089	675336±379958	1171401±318304
18	Curcumene	644-30-4	1475	1482	918343±175961	1392155±165283	399199±129983	682346±139520
19	4a,8-Dimethyl-2-(prop-1-en-2-yl)-1,2,3,4,4a,5,6,7-octahydronaphthalene	103827-22-1	1492	1476	164311±46483	161558±63842	86534±27942	156344±43539
20	α-Farnesene	502-61-4	1508	1506	170718±73140	404346±47775	238288±147579	335514±205962
21	β-Bisabolene	495-61-4	1509	1509	54376±13561	404694±35757	37892±16885	189224±47122
22	γ-Cadinene	39029-41-9	1513	1515	215524±54921	354359±117384	127616±55650	126662±28859
23	Calamenene	483-76-1	1524	1523	876990±162719	1723400±674914	434346±181595	527942±254401

24	α-Bisabolene	25532-79-0	1540	1542	203942±55095	511732±59990	108206±57967	231110±58058
25	α-Calacorene	21391-99-1	1542	1544	294006±70000	599983±250999	182823±105258	178684±71985
26	Cetene	629-73-2	1587	1589	66966±14472	94974±12815	74298±24538	77972±10280
27	α-Corocalene	20129-39-9	1629	1624	16120±6378	35165±16273	11632±7649	12208±5143
28	Neophytadiene	504-96-1	1840	1835	1231424±481070	1202868±435763	616572±334553	656986±230374
				Aldehydes				
1	(*E*)-2-Pentenal	1576-87-0	754	765	Trace	729889±403898	Trace	Trace
2	Hexanal	66-25-1	800	803	9537915±6412626	7145581±4005158	8413723±5270559	6207583±3157471
3	Furfural	98-01-1	833	830	82940367±14293092	54441004±9157127	58852137±12786102	40532191±21038710
4	(*E*)-2-Hexenal	6728-26-3	850	859	863410±248416	403722±124779	1021021±348723	585839±174014
5	(*Z*)-4-heptenal	6728-31-0	903	901	1086542±338599	639846±218121	1129403±348300	673402±250434
6	Heptanal	111-71-7	902	901	3387222±977587	1927651±743046	3183045±832952	1699512±766308
7	(*Z*)-2-heptenal	57266-86-1	958	957	2010414±787382	1373413±241130	1553138±297194	1728329±529341
8	Benzaldehyde	100-52-7	964	961	36116930±4564433	25314471±2547197	34555296±4118774	27265339±3691862
9	5-Methyl furfural	620-02-0	969	963	40155151±6347542	25259485±2579787	27922245±7482019	26300786±7297696
10	Octanal	124-13-0	1005	1004	209438±84526	124635±37268	195606±48471	147328±43128
11	(*E*,*E*)-2,4-heptadienal	4313-03-5	1012	1011	65975706±12788186	57696262±6596707	63060598±17595117	56045706±19770339
12	Benzeneacetaldehyde	122-78-1	1045	1044	2659335±596911	1717706±499435	2523342±669450	1291390±465197
13	(*E*)-2-Octenal	2548-87-0	1058	1058	5695886±1429357	4089691±786997	4768282±865798	3451390±675548
14	Nonanal	124-19-6	1102	1103	2565846±684359	3315755±1000998	3447796±1797178	5466804±3533027
15	α-Cyclociral	432-24-6	1123	1117	369045±121286	172280±54421	275195±102630	130234±37093
16	(*E*,*Z*)-2,6-Nonadienal	557-48-2	1155	1150	803460±175519	474748±91768	725849±136506	713134±105045
17	(*E*)-2-Nonenal	18829-56-6	1162	1159	1261396±203361	932431±152350	1001793±178866	840876±105076
18	Safranal	116-26-7	1201	1199	841803±100340	576372±62047	881299±290401	869796±207416
19	Decanal	112-31-2	1214	1203	513464±147917	462392±124086	413629±73479	392074±138984
20	(*E*,*E*)-2,4-Nonadienal	5910-87-2	1216	1210	1831769±528627	2031137±394691	1659091±328137	1900331±547543
21	β-Cyclocitral	432-25-7	1222	1220	6011772±736208	4192206±650601	4562672±1118498	4117602±546750
22	(*E*)-2-Decenal	3913-81-3	1259	1259	1198272±460920	1602101±294529	871492±265482	1620194±367535

23	α-Citral	141-27-5	1273	1269	964924±236087	928763±143224	827539±283472	1633548±735956
24	2-Butyl-2-octenal	13019-16-4	1378	1372	81021±26740	79737±17272	82569±32251	73241±22466
25	Dodecanal	112-54-9	1411	1409	196313±52078	143436±60229	132286±75584	137300±50461
				Ketones				
1	3-Hexanone	589-38-8	786	763	217213±114358	115698±105307	176294±106888	158741±115668
2	2-Heptanone	110-43-0	900	897	7976596±2447193	4019516±1430487	6266293±1646311	4458733±1848938
3	Methylheptenone	110-93-0	986	988	7817061±1797657	6439102±1503572	6514761±2226331	7143672±1544458
4	2,2,6-Trimethyl-cyclohexanone	2408-37-9	1047	1036	4792022±1242488	3046764±686388	3574003±1062261	3945926±837730
5	3-Octen-2-one	1669-44-9	1040	1035	881259±378066	572133±184090	920690±325149	575780±250274
6	Acetophenone	98-86-2	1065	1062	6186092±828302	4489402±615453	4553350±904219	4846653±470638
7	(*E,E*)-3,5-Octadien-2-one	30086-02-3	1073	1070	17009454±7291236	13034552±2284962	16921489±5377339	12066580±3490242
8	2-Nonanone	821-55-6	1095	1091	2422811±634646	1245200±185988	1880900±368813	1836803±566113
9	3,5-Octadien-2-one	38284-27-4	1091	1093	4485531±1603511	3417132±297366	5274929±1268578	4871113±739469
10	6-Methyl-3,5-heptadiene-2-one	1604-28-0	1107	1104	1778729±305990	1685573±275387	1973638±635755	2710301±586368
11	3-Nonen-2-one	14309-57-0	1136	1138	477011±83337	387633±69332	489473±78825	385941±92697
12	Ketoisophorone	1125-21-9	1147	1144	281270±51608	183562±29598	236777±53497	289280±62248
13	5-Ethyl-6-methyl-3E-hepten-2-one	57283-79-1	1144	1147	2261912±457272	1813806±348237	2095307±470529	2176125±526778
14	Propiophenone	93-55-0	1176	1165	10245321±2206781	7391195±1693691	9074286±4557219	9112156±2225084
15	3'-Methylacetophenone	585-74-0	1176	1173	Trace	1056449±114352	Trace	1332171±208150
16	*p*-Methylacetophenone	122-00-9	1183	1184	7576313±1750795	5977933±733531	5353030±1385632	7133400±1244885
17	6-Dodecanone	6064-27-3	1354	1369	225606±44260	219186±28743	194769±56750	252025±71410
18	*cis*-Jasmone	488-10-8	1391	1398	256552±60273	335853±52164	177218±64644	252157±79520
19	6,10-Dimethyl-2-undecanone	1604-34-8	1407	1402	1000036±193074	888282±127676	898318±223783	1138688±285324
20	α-Ionone	127-41-3	1427	1427	3695045±942317	2980802±512329	3777698±1491687	3555107±615775
21	Dihydro-β-ionone	17283-81-7	1433	1438	110308±19348	58188±13766	77392±20467	65794±20913
22	Geranylacetone	3796-70-1	1454	1451	1531311±347751	1223186±246841	1379381±532041	1277463±280042
23	2,6-Bis(1,1-dimethylethyl)-2,5-cyclohexadiene-1,4-dione	719-22-2	1458	1466	60504±13654	53561±17746	68339±11296	68082±25178
24	Dehydro-β-ionone	1203-08-3	1485	1482	849429±288923	644995±130093	911377±525379	894541±381567

25	*trans*-β-Ionone	79-77-6	1485	1486	13131104±3560214	12332298±2899648	13368337±5714523	14831433±2732967
26	Benzophenone	119-61-9	1621	1627	11856±3017	9215±2358	27890±15504	19590±9531
27	Hexahydrofarnesyl acetone	502-69-2	1848	1841	1335293±186322	1432912±184999	1361761±372807	1535245±282768
				Nitrogen compounds				
1	2-Furonitrile	617-90-3	822	805	387039±76064	311855±52604	238082±89476	194122±105510
2	*N*-Ethylpyrrole	617-92-5	821	813	8984454±5468682	5023099±5353637	8483378±7064660	2906451±2254040
3	2-Methylpyrazine	109-08-0	831	815	5903033±1791165	3412199±1085723	6003749±3548791	2849275±926861
4	2,5-Dimethylpyrazine	123-32-0	917	901	7997872±2611541	3688585±1189209	5640389±1777914	4623914±1187735
5	Ethylpyrazine	13925-00-3	917	916	2435972±973922	1230820±194011	2271540±1110964	1362923±702279
6	1-Ethyl-1H-pyrrole-2-carboxaldehyde	2167-14-8	1046	1050	43724014±4269388	25890041±5160970	39389332±10870472	30165411±6515311
7	2-Acetylpyrrole	1072-83-9	1059	1063	2058446±1653284	1487317±683882	1825766±871208	2526568±1108169
8	3-Ethyl-2,5-dimethylpyrazine	13360-65-1	1082	1081	2957006±332950	1747712±346924	3326293±1263091	2010274±537240
9	Benzyl nitrile	140-29-4	1140	1140	36894805±5208460	50686793±5656634	24440090±8151536	34378558±8451279
10	2-Methyl-3,5-diethylpyrazine	18138-05-1	1162	1155	1247466±126107	800001±187041	1367352±569980	904487±305321
11	1-Furfurylpyrrole	1438-94-4	1185	1182	5152867±532808	3657326±1281901	3697424±1398568	1861022±975544
12	2,5-Dimethyl-3-(2-methylpropyl)-pyrazine	32736-94-0	1193	1200	1860604±146255	1103204±174104	1884757±565231	1143795±337554
13	Indole	120-72-9	1295	1291	4988972±1298004	8329810±2859921	4117926±2234708	5539731±3245720
14	(2-Nitroethyl)-benzene	6125-24-2	1300	1298	3038323±976285	8374927±1074855	1694627±1217736	3701489±1329787
15	2,5-Dimethyl-3-(2-methylbutyl)pyrazine	72668-36-1	1308	1302	721124±136785	396035±52062	761303±261776	496162±151916
16	Caffeine	58-08-2	1842	1846	9876932±10626121	10562311±6526341	30259756±70809033	15504358±9198878
				Alcohols				
1	1-Pentanol	71-41-0	764	773	618070±628315	627138±218929	903216±572583	663187±466960
2	1-Hexanol	111-27-3	865	874	730018±363884	432146±228206	785070±310249	720823±324212
3	1-Octen-3-ol	3391-86-4	983	979	714934±196225	531118±121724	708200±200917	703786±164512
4	(*Z*)-2-Octen-1-ol	26001-58-1	1066	1067	Trace	Trace	Trace	108312±44134
5	*trans*-Linalool oxide (furanoid)	34995-77-2	1067	1086	6126530±1102874	7803882±1946432	4379508±1107546	7776820±1163475
6	Linalool	78-70-6	1101	1099	2651240±556716	2342978±433003	2610576±1293723	3931313±1158837
7	Phenylethyl alcohol	60-12-8	1116	1114	6875926±1970781	14018828±4179364	4283542±2123792	8696286±1974443

8	*trans*-Linalool oxide (pyranoid)	39028-58-5	1173	1170	555313±64613	499671±69211	464322±93569	572770±122205
9	α-Terpineol	98-55-5	1198	1191	2574724±607119	1592411±241439	1935018±376012	2027137±632847
10	Geraniol	106-24-1	1255	1253	1992704±520538	1868495±333316	3034032±2523911	6414409±4015219
11	(*Z*)-Nerolidol	142-50-7	1535	1531	43433±20983	51508±16035	96167±231389	66019±12334
12	(*E*)-Nerolidol	40716-66-3	1564	1563	7126252±1553684	12717970±1301714	5113156±3182824	7885211±2170168
13	Cedrol	77-53-2	1596	1605	118173±27153	217143±214760	146106±65999	249578±202805
14	α-Bisabolol oxide B	26184-88-3	1658	1656	449001±137752	408129±108133	326844±150360	546216±126185
15	Isophytol	505-32-8	1939	1943	302204±65243	329633±52539	293212±125173	202064±119167
16	Phytol	150-86-7	2114	2106	44285±17776	36932±17290	83023±94583	36751±12648
				Aromatic compounds				
1	Toluene	108-88-3	766	775	11401874±8860234	7188358±3660632	6625037±3423587	7336697±1970753
2	*m*-Xylene	108-38-3	866	868	28202235±13299611	19856179±4094103	10147671±6787252	19196597±6254790
3	*o*-Ethyl toluene	611-14-3	972	982	166782±39878	158722±36390	158713±43481	139925±32647
4	Mesitylene	108-67-8	995	996	8736325±1841623	5907706±904682	6904704±1675275	7873297±1112978
5	*p*-Cymene	99-87-6	1018	1026	20847492±5547935	17507725±3997249	13644994±6245684	19014260±5843075
6	Naphthalene	91-20-3	1182	1183	2917483±339649	3668207±378357	6605726±3526188	4289079±1190928
7	1,3-Bis(1,1-dimethylethyl)-benzene	1014-60-4	1247	1253	10904867±3209232	12470755±1846738	10183379±5072495	12928910±4283242
8	4-(1-Methylpropyl)-phenol	99-71-8	1314	1318	219507±128623	149977±29637	246844±128873	253166±49193
9	1,1,6-Trimethyl-1,2-dihydronaphthalene	30364-38-6	1354	1353	35215404±6275931	25897624±3490935	21981098±4794929	22218986±4895061
10	2,2',5,5'-Tetramethylbiphenyl	3075-84-1	1663	1678	35741±12785	50240±57726	70775±17703	70897±70393
11	Phenanthrene	85-01-8	1775	1775	80975±28003	134397±22371	117765±78398	116527±29722
				Oxygen heterocyclic compounds				
1	2-Propylfuran	4229-91-8	792	794	138656±71567	129096±140369	141558±75576	66815±34890
2	2-(2-Propenyl)-furan	75135-41-0	856	851	Trace	273150±83971	Trace	Trace
3	2-Butylfuran	4466-24-4	897	900	871649±275277	498166±174856	888544±290507	479862±152967
4	2-Acetylfuran	1192-62-7	911	903	8372669±1443655	5477618±1082179	8208251±2699465	4976745±1199095
5	2,2,6-Trimethyl-6-vinyltetrahydropyran	7392-19-0	972	976	164544±47351	121461±31603	111916±34769	234669±76030
6	2-Pentylfuran	3777-69-3	991	993	3442164±775129	2147598±691185	3353326±1311395	1682230±543209

7	*trans*-Anhydrolinalool oxide (furanoid)	54750-70-8	994	995	514963±144554	428330±129672	338248±95484	396104±82272
8	*cis*-Anhydrolinalool (furanoid)	54750-69-5	1009	1010	389551±128957	364333±130393	239760±70486	302149±64852
9	2,2'-Methylenebis-furan	1197-40-6	1086	1103	951276±409157	390551±107285	429891±166053	292940±110272
10	*trans*-Rose oxide	5258-11-7	1127	1129	Trace	Trace	Trace	102332±50346
11	Dibenzofuran	132-64-9	1521	1513	79112±28789	132068±24990	125641±56125	191652±69103
				Lactones				
1	2(5H)-Furanone	497-23-4	916	926	373308±131007	291143±66670	333213±186395	351313±97339
2	γ-Nonanolactone	104-61-0	1363	1361	1749056±414387	1944817±239073	1357099±385879	1893960±708716
3	Coumarin	91-64-5	1432	1436	193714±87592	256193±71451	156997±65949	1046515±381376
4	Dihydroactinidiolide	17092-92-1	1525	1532	7714314±2571100	6288639±1589075	7898098±3296949	9763377±2396255
				Sulfur compounds				
1	Dimethyl disulfide	624-92-0	746	760	485007±318850	146460±109442	250247±156057	103753±77587
2	2-Methylthiophene	554-14-3	787	779	178727±119576	139453±37169	148684±56389	65672±53060
3	Dimethyl trisulfide	3658-80-8	970	970	60481±12525	16844±6657	55007±13890	28125±3259
				Organic acids				
1	4-Hydroxybutyric acid	591-81-1	933	923	261038±83880	158090±41852	186084±53497	211266±75898

[a]: The retention index (RI) values were the reference values for DB-5MS (30 m×250 μm×0.25 μm), HP-5MS (30 m×250 μm×0.25 μm) or other columns with a similar stationary phase under the similar gas chromatography (GC) conditions, and the values were obtained from NIST 2014; [b]: RI: retention index; the RI values were calculated from C8-C40 n-alkanes.

Table S2. The detailed panel performance on the gas chromatography olfactometry (GC-O) results of Dahongpao (DHP).

No	Compound	Detailed panel performance											
		Panelists 1		Panelists 2		Panelists 3		Panelists 4		Panelists 5		Panelists 6	
		Aroma characteristics	AI [a]	Aroma characteristics	AI	Aroma characteristics	AI	Aroma characteristics	AI	Aroma characteristics	AI	Aroma characteristics	AI
1	Hexanal	Fresh	1	Green	3	Grassy	3	Fresh	1	Green	3	Grassy	3
2	*N*-Ethylpyrrole	Burnt	3	/	-	Baked	4	/	-	/	-	Pungent, baked	2
3	2-Methylpyrazine	Roasted, chocolate	2	/	-	Cocoa, roasted	3	/	-	Roasted, nutty	2	Pungent, pesticide-like	2
4	Heptanal	Fatty, herbal	3	Fatty, green	3	Green, fatty	4	Fatty, herbal	2	Fatty	3	Fatty	2
5	2,5-Dimethylpyrazine	Coffee	2	Coffee, fatty	4	Baked, chocolate	4	Baked, woody	3	Roasted, nutty	3	Coffee, roasted nuts	3
6	2-Acetylfuran	Cocoa, roasted nuts	3	Roasted bread, nutty	3	Chocolate	3	Cocoa, caramel	3	/	-	Roasted, coffee	3
7	Dimethyl trisulfide	Pungent, sulfurous	1	/	-	/	-	/	-	Sulfurous, pickled	3	Pickled, fatty	3
8	1-Octen-3-ol	Mushroom, earthy	2	Earthy, fresh	3	/	-	Green, fatty	2	Mushroom, rusty	3	/	-
9	Methylheptenone	Citrus	2	Green	4	Floral	2	Green	3	Green	3	Green ,lemon	3
10	Octanal	/	-	/	-	Fruity, fatty	2	Green	2	Orange, green	2	/	-
11	(*E*,*E*)-2,4-heptadienal	Green, slight pungent	2	Slight pungent	-	Fatty, green	2	/	-	/	-	/	-
12	*p*-Cymene	Fresh, sweet	2	Fragrant and sweet	4	Fragrant and sweet	4	Rice-like, sweet	3	/	-	Fresh, citrus	3
13	2,2,6-Trimethyl-cyclohexanone	/	-	Honey, cooked rice	4	/	-	/	-	/	-	/	-
14	3-Octen-2-one	Herbal	2	Herbal, fruity	4	/	-	Fresh, fruity	3	Herbal	2	Herbal, sweet	3
15	Benzeneacetaldehyde	Green, floral	-	Rose-like	4	Rose-like	3	Rose-like	4	Floral, green	3	Rose-like	3
16	*trans*-β-Ocimene	/	-	Herbal, floral	3	Floral, sweet	3	Floral	4	Floral	2	Herbal	3
17	(*E*)-2-octenal	Green	3	Green, herbal	4	/	-	Green, nutty	2	Green, leaf	3	Green	3
18	Acetophenone	/	-	Floral	3	Sweet	3	Sweet, floral	4	Sweet	3	Sweet	4
19	2-Acetylpyrrole	/	-	/	-	Caramel	3	Caramel, sweet	3	Sweet	3	/	-
20	*trans*-Linalool oxide (furanoid)	Floral	2	Sweet	3	/	-	/	-	/	-	Floral, sweet	4
21	3-Ethyl-2,5-dimethylpyrazine	/	-	Roasted, coffee	3	Roasted, caramel	3	/	-	Coffee	3	Roasted, nut	3
22	2,2'-Methylenebis-furan	Roasted	3	Rich roasted, nut	4	Roasted, caramel	2	Roasted, bitter	2	Roasted, coffee	3	Roasted, bitter	3
23	3,5-Octadien-2-one	/	-	Mushroom-like	4	Mushroom	3	Mushroom, bitter	2	Mushroom, fruity	3	Mushroom, fatty	3
24	Methyl benzoate	/	-	leather	2	Floral, phenolic	3	/	-	Floral, leather	2	Floral	3
25	Linalool	Floral, fresh	2	Floral, sweet	3	Floral, citrus	2	Sweet	2	Floral	4	Floral	4

26	Nonanal	Floral	2	Floral, fruity	4	Floral, fresh	2	Floral, fresh	2	Floral, sweet	4	Floral, fresh	3
27	6-Methyl-3,5-heptadiene-2-one	/	-	Sweet, floral	3	Coconut creamy	3	Sweet, creamy	3	/	-	/	-
28	Phenylethyl Alcohol	Floral	3		-	Rose, sweet	3	Sweet, fresh	2	Floral	3	Floral, rose-like	3
29	Methyl octanoate	Fruity, woody	3	sweet, green	4	Fruity, fresh	2	Green	3	/	-	Sweet, green	4
30	*trans*-Alloocimene	Green, woody	2	Green, fresh	4	Floral	3	/	-	Green, herbal	3	Fresh, floral	4
31	5-Ethyl-6-methyl-3E-hepten-2-one	/	-	Green	3	Green	1	Green	3	Green, fresh	3	Fresh, floral	3
32	(*E*,*Z*)-2,6-Nonadienal	Green, fresh	3	Cucumber	4	Cucumber	3	Green, herbal	3	Green, leaf	3	Green	3
33	2-Methyl-3,5-diethylpyrazine	/	-	Nutty	4	Nutty	3	/	-	Green, nutty	3	Green	3
34	(*E*)-2-nonenal	Green, aldehydic	2	/	-	Green, cucumber	3	Green, bitter	2	Green, fresh	3	Green, leaf	3
35	Benzyl acetate	/	-	/	-	Green, fresh	3	Fresh, sweet	2	Green, floral	3	Green, fresh	3
36	Propiophenone	/	-	/	-	Green, herbal	3	Fresh	2	Green, leaf	3	Green, fresh	3
37	Naphthalene	Pungent	2	Pungent	3	Pepper, earthy	2	Pungent	1	Pungent	4	Empyreumatic	4
38	1-Furfurylpyrrole	/	-	Roasted, plastic	3	Green	3	Green	1	/	-	Roasted, herbal	4
39	Methyl salicylate	Minty	2	Wintergreen，green	3	Wintergreen-like	2	/	-	Minty, herbal	4	Wintergreen, herbal	4
40	Safranal	Metallic, leaf	2	Herbal, fresh	3	Herbal	2	/	-	Herbal, metallic	3	/	-
41	(*E*,*E*)-2,4-Nonadienal	Green, vegetable	3	Green, sweet	4	Green	3	Green, fruity	2	Green, plastic	3	Green, fresh	4
42	Phenethyl acetate	/	-	Floral, fresh	3	Cucumber, green	3	Floral, sweet	2	Floral	3	Floral, cucumber	3
43	(*E*)-2-decenal	Green	2	Green, earthy	2	Fresh	3	/	-	/	-	Green, leaf	3
44	α-citral	Citrus, lemon	2	/	-	Citrus, fresh	2	/	-	Fresh	2	/	-
45	Indole	/	-	Fresh, floral	3	/	-	/	-	Fresh, floral	3	Floral, leather	3
46	γ-Nonanolactone	/	-	Coconut creamy	3	Sweet, floral	1	Creamy	1	Floral	2	Floral, sweet	3
47	Hexyl hexanoate	Floral	2	Floral, fruity	2	/	-	/	-	Fruity, herbal	1	Honey, floral, fruity	3
48	(*E*)-2-Hexenyl hexanoate	Fruity, clean	3	/	-	Floral, sweet	3	/	-	Fruity, floral, sweet	2	/	-
49	α-Cedrene	Floral, sweet	2	Floral, fruity	3	Floral, sweet	2	/	-	Floral, sweet, fruity	2	/	-
50	α-Ionone	Floral	2	Floral	-	Sweet, creamy	2	Floral	3	Floral, sweet	2	Coconut milk, sweet	3
51	Coumarin	/	-	/	-	/	-	/	-	/	-	/	-
52	Phenethyl butyrate	/	-	Floral, fruity	2	Floral, sweet	2	/	-	/	-	Floral, vanilla	4
53	(*E*)-β-Famesene	/	-	Floral, herbal	3	Floral, sweet, woody	1	Floral, grass	2	/	-	Sweet	2
54	*trans*-β-Ionone	Rose-like	3	/	-	Floral, rose-like	3	Coconut creamy	3	/	-	Floral, creamy	2
55	Calamenene	/	-	/	-	Fresh	2	Fresh and cool	2	Fresh, mint	2	Fresh and cool	2
56	Dihydroactinidiolide	/	-	Essential oil	2	Herbal, minty	2	/	-	Herbal, fresh	2	Herbal, clean	2

[a]: AI: aroma intensity.

Table S3. The detailed panel performance on the gas chromatography olfactometry (GC-O) results of Rougui (RG).

No	Compound	Detailed panel performance											
		Panelists 1		Panelists 2		Panelists 3		Panelists 4		Panelists 5		Panelists 6	
		Aroma characteristics	AI	Aroma characteristics	AI	Aroma characteristics	AI	Aroma characteristics	AI	Aroma characteristics	AI	Aroma characteristics	AI
1	Hexanal	Green, leafy	3	Green, fresh	3	Grassy	2	/	-	/	-	Grassy	3
2	*N*-Ethylpyrrole	/	-	Burnt	2	Roasted nuts	3	Burnt	2	Burnt, bitter	3	Pungent, baked	3
3	2-Methylpyrazine	/	-	Roasted, bitter	4	Pungent, roasted	3	Pungent, pesticide	2	Roasted, nutty	2	/	-
4	Heptanal	Fatty, green	1	Fatty, herbal	3	Green, herbal	3	Green, fatty	1	Fatty	2	Fatty, grassy	2
5	2,5-Dimethylpyrazine	Baked, chocolate	3	Coffee	4	/	-	Baked, bitter	2	Roasted	2	Coffee	3
6	2-Acetylfuran	/	-	Roasted bread	4	Chocolate	3	Baked, cocoa	2	Cocoa	2	Roasted, cocoa	3
7	Dimethyl trisulfide	Sulfurous	1	/	-	Pungent	3	Pickled, pesticide	1	Sulfurous	2	Pickled, rusty	3
8	1-Octen-3-ol	/	-	Earthy, mushroom	4	Mushroom, rusty	3	Green, fresh	3	Mushroom	3	Fresh, earthy	2
9	Methylheptenone	Citrus, lemon	2	Green, fresh	3	Green, fresh	3	Floral, lemon	3	Green, floral	2	Green ,lemon	3
10	Octanal	/	-	Green	3	Green, fruity, fatty	3	/	-	/	-	Green, fruity	3
11	(*E*,*E*)-2,4-heptadienal	Green, vegetable	3	/	-	Green	4	Fatty, pungent	3	/	-	Fatty, green	3
12	*p*-Cymene	Fresh	1	Fragrant and sweet	4	Fragrant and sweet	3	Fresh, cooked rice	1	Rice-like	3	/	-
13	2,2,6-Trimethyl-cyclohexanone	/	-	/	-	/	-	/	-	/	-	/	-
14	3-Octen-2-one	Herbal	2	Herbal, fruity	4	Herbal, sweet	3	Fresh, fruity	2	/	-	Herbal	3
15	Benzeneacetaldehyde	/	-	Floral, rose	4	Rose-like	3	Green, rose	2	Green, floral	3	Rose-like, sweet	3
16	*trans*-β-Ocimene	Floral, herbal	2	/	-	Floral, sweet	4	/	-	Floral, herbal	3	/	-
17	(*E*)-2-octenal	/	-	/	-	Green, fresh	3	/	-	Fresh, nut	2	Green, nut	4
18	Acetophenone	Sweet, floral	2	Floral	4	Sweet	4	Sweet, floral	2	Sweet	3	Sweet, floral	4
19	2-Acetylpyrrole	/	-	Caramel, sweet	1	Caramel	4	/	-	/	-	Caramel	4
20	*trans*-Linalool oxide (furanoid)	/	-	Floral, sweet	3	Floral, sweet	4	Floral	2	Floral, sweet	3	Floral, sweet	4
21	3-Ethyl-2,5-dimethylpyrazine	Roasted	2	Coffee	2	/	-	Roasted, bitter	2	Coffee	3	Roasted, nutty	3
22	2,2'-Methylenebis-furan	Roasted	2	Roasted, bitter	4	Roasted, pungent	3	Roasted, bitter	3	Nutty, bitter	3	Roasted	3
23	3,5-Octadien-2-one	/	-	Mushroom, fruity	4	/	-	Mushroom	1	Earthy, fresh	3	Mushroom, fatty	3
24	Methyl benzoate	Floral, fresh	2	Green, leather	4	Floral	3	Wintergreen, phenolic	1	Floral, leather	4	Floral	3
25	Linalool	Floral	3	Floral, sweet	4	Floral	2	Sweet	3	Floral, milky	3	Floral	3

26	Nonanal	Floral	2	/	-	Floral, fresh	3	Floral, fresh	3	Floral, butter	3	Floral, fresh	4
27	6-Methyl-3,5-heptadiene-2-one	/	-	Sweet, spice	3	Coconut creamy	3	/	-	Sweet, creamy	3	/	-
28	Phenylethyl alcohol	Floral, honey	3	Floral, rose	3	Sweet	3	/	-	Floral	3	Floral, sweet	4
29	Methyl octanoate	Fruity, woody	2	sweet, green	3	Fruity, fresh	3	Green	3	Fruity, fresh	3	Sweet, green	4
30	*trans*-Alloocimene	/	-	Green, fresh	4	Floral, fresh	3	/	-	Green, grassy	3	Fresh, floral	3
31	5-Ethyl-6-methyl-3E-hepten-2-one	/	-	Green, fresh	3	/	-	Green	3	Green, fresh	3	Green	1
32	(*E*,*Z*)-2,6-Nonadienal	Green, fresh	2	Cucumber	4	Cucumber	4	Green, grassy	2	Green, leaf	3	Green	2
33	2-Methyl-3,5-diethylpyrazine	Green, vegetable	1	/	-	/	-	/	-	/	-	/	-
34	(*E*)-2-nonenal	/	-	Green, plastic	3	Green, cucumber	3	Green	2	Cucumber, fresh	3	Green, leaf	4
35	Benzyl acetate	Fresh, fruity	3	/	-	Green	4	Fresh, cucumber	2	Green	3	Green, fresh	2
36	Propiophenone	/	-	Green, fresh	3	Green	2	Fresh	2	Green	3	Green, fresh	4
37	Naphthalene	Pungent	4	Pungent, earthy	4	Pungent	3	Earthy, burnt	1	/	-	Peppery	3
38	1-Furfurylpyrrole	Roasted	1	Roasted, plastic	3	Green, pungent	4	/	-	Roasted, green	3	/	-
39	Methyl salicylate	/	-	Wintergreen，herbal	3	/	-	Minty	3	Minty, herbal	3	Wintergreen-like	3
40	Safranal	Metallic	2	/	-	/	-	Herbal	2	Herbal, metallic	2	Metallic, leaf	2
41	(*E*,*E*)-2,4-Nonadienal	/	-	Green, melon	4	Green	4	Fruity	2	Green	3	Green, fruity	3
42	Phenethyl acetate	/	-	Floral, sweet	4	Cucumber, milky	4	Floral, fruity	2	Floral, sweet	4	Floral, cucumber	3
43	(*E*)-2-decenal	Green	1	Green, milky	3	Fresh	4	/	-	Green, fresh	3	Green	3
44	α-citral	Citrus, herbal	2	/	-	Citrus, fresh	2	/	-	Fresh, woody	3	/	-
45	Indole	/	-	/	-	Smelly, leather	3	Floral	1	Fresh, floral	2	Fresh, floral	4
46	γ-Nonanolactone	Floral	1	Creamy	3	Sweet, creamy	3	Coconut, sweet	2	Coconut creamy	3	Floral, sweet	3
47	Hexyl hexanoate	Floral, sweet	3	Floral, honey	3	Grass, fruity	1	/	-	Fruity, floral	3	Fruity, floral	4
48	(*E*)-2-Hexenyl hexanoate	Fruity, clean	2	/	-	Fruity, sweet	2	/	-	Floral, sweet	3	Fruity, floral	4
49	α-Cedrene	/	-	Floral, fruity	3	Floral, sweet, fruity	3	Floral, sweet	3	Floral, sweet	3	Floral, sweet	2
50	α-Ionone	Floral, sweet	2	Floral, fresh	2	Sweet	3	Floral, milk-like	2	/	-	/	-
51	Coumarin	Floral, sweet	2	Milk-like, sweet	4	Milk-like, sweet	3	/	-	Sweet	2	Floral, milk-like, sweet	3
52	Phenethyl butyrate	Fresh, floral	2	Fruity	4	Sweet, fruity	3	/	-	Floral, sweet	2	Floral, fruity	3
53	(*E*)-β-Famesene	Floral, fresh	2	Sweet, milky	4	Sweet	3	Floral, fruity	2	Floral	3	Sweet, herbal	2
54	*trans*-β-Ionone	Rose-like	3	/	-	Rose-like	3	Coconut creamy	3	Coconut creamy	2	Floral, creamy	3
55	Calamenene	/	-	Fresh and cool	3	/	-	/	-	Fresh, minty	3	Fresh and cool	3

56	Dihydroactinidiolide	/	-	/	-	Herbal, essential oil	1	/	-	Herbal, clean	3	Herbal, fresh	2

Table S4. The detailed panel performance on the gas chromatography olfactometry (GC-O) results of Shuixian (SX).

No	Compound	Detailed panel performance											
		Panelists 1		Panelists 2		Panelists 3		Panelists 4		Panelists 5		Panelists 6	
		Aroma characteristics	AI	Aroma characteristics	AI	Aroma characteristics	AI	Aroma characteristics	AI	Aroma characteristics	AI	Aroma characteristics	AI
1	Hexanal	Grassy	1	/	-	/	-	Green, fresh	1	Green	3	Grassy	2
2	*N*-Ethylpyrrole	Burnt	3	/	-	/	-	Baked	3	Burnt, bitter	2	Burnt, pungent	4
3	2-Methylpyrazine	Pungent, roasted	3	/	-	Baked peanut	3	Pesticide-like	2	Roasted	2	/	-
4	Heptanal	Fatty, green	1	Fatty, herbal	4	Fatty, herbal	-	Green, fatty	2	Herbal, fatty	4	Grassy	2
5	2,5-Dimethylpyrazine	Coffee	2	Coffee	3	Baked	2	/	-	Baked nutty	2	Roasted bread	3
6	2-Acetylfuran	Coffee	3	Roasted bread	3	Cocoa	3	Roasted	4	Roasted	2	Roasted bread	3
7	Dimethyl trisulfide	Sulfurous	1	Pungent	3	Sulfurous, pesticide	3	Pickled, pungent	1	Sulfurous	3	Pickled, metallic	3
8	1-Octen-3-ol	Herbal, fresh	1	Earthy	3	Woody, fresh	3	Mushroom, herbal	3	Mushroom	3	Fresh	3
9	Methylheptenone	Lemon	2	Fresh, floral	3	Green, fatty	3	Floral, lemon	1	Green	4	Green, lemon	3
10	Octanal	Fruity, fresh	2	/	-	/	-	Green, fatty	1	Green, fruity	3	/	-
11	(*E*,*E*)-2,4-heptadienal	/	-	Green, Fatty	4	Green, pungent	4	Vegetable, fatty	3	/	-	/	-
12	*p*-Cymene	Rice crust	1	Fragrant, fresh	3	/	-	Fresh, cooked rice	2	Fragrant and sweet	2	/	-
13	2,2,6-Trimethyl-cyclohexanone	/	-	/	-	/	-	/	-	/	-	/	-
14	3-Octen-2-one	Herbal, earthy	3	/	-	/	-	Fresh, fruity	2	Fruity	4	Herbal	3
15	Benzeneacetaldehyde	/	-	Floral, green	3	Rose-like	3	Green, rose	3	Floral	3	Green, herbal	3
16	*trans*-β-Ocimene	/	-	Herbal	3	Floral	3	/	-	Floral, herbal	3	Herbal	3
17	(*E*)-2-octenal	Green	3	/	-	Green, fresh	3	Green	4	Nutty	3	Green	3
18	Acetophenone	Sweet	3	Sweet, floral	3	Caramel	3	Sweet, floral	3	Sweet, blueberry	3	Sweet, floral	4
19	2-Acetylpyrrole	/	-	Caramel	3	Caramel, sweet	3	/	-	/	-	Caramel	3
20	*trans*-Linalool oxide (furanoid)	Sweet	2	Floral, sweet	4	Floral	3	/	-	Sweet	3	Earthy, sweet	3
21	3-Ethyl-2,5-dimethylpyrazine	Roasted	2	Coffee, bitter	3	Coffee	4	Roasted, nutty	3	Coffee	3	Coffee	4
22	2,2'-Methylenebis-furan	Roasted	1	Roasted	4	Coffee, bitter	2	Nutty, bitter	2	Coffee, roasted	3	/	-
23	3,5-Octadien-2-one	/	-	Herbal, mushroom	4	/	-	Fatty	2	Earthy, mushroom	3	Mushroom, sweet	3
24	Methyl benzoate	/	-	Leather	4	/	-	Floral, fresh	2	Floral, leather	3	Floral, herbal	3
25	Linalool	Floral	3	Floral, sweet	4	Floral	2	Sweet	2	Floral, milky	3	/	-
26	Nonanal	Floral	2	Fatty	3	Floral, fruity	2	Fresh	3	Floral, caramel	4	Floral, fresh	3

27	6-Methyl-3,5-heptadiene-2-one	Rice-like	3	/	-	Coconut, sweet	3	Sweet	3	Coconut creamy	3	/	-
28	Phenylethyl alcohol	Floral, fruity	2	/	-	Floral, sweet	3	Floral, fresh	2	Sweet	3	Sweet, honey	2
29	Methyl octanoate	Fruity, fresh	3	/	-	Fruity, green	3	Floral, fruity, fresh	3	Sweet	3	Sweet, honey	3
30	trans-Alloocimene	/	-	Green	3	Green, fresh	3	Fresh, floral	3	Green, grassy	3	Fresh, floral	4
31	5-Ethyl-6-methyl-3E-hepten-2-one	Acidulous, fresh	2	Cucumber, fresh	3	Green, grassy	2	Green	3	Floral, fresh	3	Green, fresh	3
32	(*E,Z*)-2,6-Nonadienal	Cucumber, fresh	3	Fresh	4	Green apple	3	Green	3	Cucumber, fresh	4	Green, grassy	3
33	2-Methyl-3,5-diethylpyrazine	/	-	Nutty, vegetable	4	/	-	Nutty, green	2	Coffee, nutty	4	/	-
34	(*E*)-2-nonenal	/	-	Green	4	/	-	Green	2	Cucumber, fresh	3	Green, cucumber	3
35	Benzyl acetate	/	-	/	-	Green	4	Fresh, fruity	1	Fresh	3	Green, fresh	3
36	Propiophenone	/	-	/	-	Green	4	Green, fresh	1	Fresh	3	Green	3
37	Naphthalene	Pungent	3	Pungent, earthy	3	Earthy	4	Earthy, burnt	3	Earthy	4	Pungent	3
38	1-Furfurylpyrrole	Green, pungent	2	Roasted	3	Pungent, plastic	4	Roasted	1	Green, pesticide	3	/	-
39	Methyl salicylate	/	-	/	-	/	-	Minty, herbal	2	Minty	3	Wintergreen, herbal	3
40	Safranal	/	-	/	-	Herbal, fresh	2	Green, metallic	3	Green peas, rusty	2	/	-
41	(*E,E*)-2,4-Nonadienal	Fruity	2	Green, melon	4	Green	4	Fruity, fresh	3	Green, salty	3	Fruity, metallic	1
42	Phenethyl acetate	/	-	/	-	/	-	Floral	3	Floral, sweet	3	Cucumber, sweet	2
43	(*E*)-2-decenal	Green	3	/	-	/	-	/	-	Green, fresh	3	Green, fresh	3
44	α-citral	Citrus, herbal	2	Fresh, earthy	2	/	-	Citrus, fresh	2	/	-	/	-
45	Indole	Floral, fresh	1	Leather	1	/	-	Floral	1	/	-	/	-
46	γ-Nonanolactone	/	-	Creamy, sweet	3	Coconut creamy	2	/	-	Coconut, sweet	2	Floral, sweet	3
47	Hexyl hexanoate	Floral, sweet	3	Floral, honey	2	Grass, fruity	2	/	2	Fruity, floral	3	Fruity, floral	3
48	(*E*)-2-Hexenyl hexanoate	/	-	/	3	Fruity, sweet	2	Fruity, floral	2	Floral, sweet	3	/	-
49	α-Cedrene	/	2	Floral, fruity	3	Floral, sweet, fruity	2	Floral, sweet	2	Floral, sweet	2	Floral, sweet	3
50	α-Ionone	Floral	2	/	-	Sweet, floral	1	/	-	Sweet, floral	2	Sweet	1
51	Coumarin	/	-	/	-	/	-	/	-	/	-	/	-
52	Phenethyl butyrate	Floral, sweet	1	Floral	3	Floral, fruity	3	/	-	Floral, sweet	2	/	-
53	(*E*)-β-Famesene	Floral	2	Sweet, milky	3	Sweet, earthy	1	/	-	/	-	/	-
54	*trans*-β-Ionone	Rose, floral	3	/	-	Rose-like	3	Coconut creamy	2	/	-	Floral, creamy	4
55	Calamenene	/	-	Fresh and cool	2	/	-	Fresh	1	Fresh and cool	3	/	-
56	Dihydroactinidiolide	Herbal, fresh	2	/	-	/	-	Herbal, fresh	1	Clean, essential oil	3	/	-

Table S5. The detailed panel performance on the gas chromatography olfactometry (GC-O) results of Jinfo (JF).

No	Compound	Detailed panel performance											
		Panelists 1		Panelists 2		Panelists 3		Panelists 4		Panelists 5		Panelists 6	
		Aroma characteristics	AI	Aroma characteristics	AI	Aroma characteristics	AI	Aroma characteristics	AI	Aroma characteristics	AI	Aroma characteristics	AI
1	Hexanal	Grassy, green	1	Grassy	3	Green, grassy	2	/	-	Green apple	3	Grassy	2
2	*N*-Ethylpyrrole	/	-	Burnt	2	/	-	/	-	Burnt	2	Burnt, baked	2
3	2-Methylpyrazine	/	-	Pungent, pesticide	4	Pesticide-like	2	/	-	Roasted	3	Roasted, burnt	2
4	Heptanal	Green, fatty	1	Herbal, fatty	4	/	-	Green, fatty	3	/	-	Herbal, grassy	3
5	2,5-Dimethylpyrazine	Coffee	2	/	-	Baked	3	/	-	Coffee	3	Baked, coffee	4
6	2-Acetylfuran	Coffee	2	/	-	Cocoa, roasted	3	/	-	Roasted, coffee	3	Coffee	3
7	Dimethyl trisulfide	Sulfurous	1	Pungent, pesticide	4	Pickled, rusty	3	Sulfurous	1	Pickled, pungent	2	Pungent, metallic	2
8	1-Octen-3-ol	Herbal	2	Earthy, mushroom	4	Fresh, herbal	2	Mushroom, herbal	2	Mushroom	3	/	-
9	Methylheptenone	/	-	Green	4	Floral, metallic	3	Floral, lemon	2	Fresh, green	4	Citrus, lemon	3
10	Octanal	Fruity	1	Green, fatty	3	/	-	/	-	/	-	Fresh, fruity	2
11	(*E*,*E*)-2,4-heptadienal	Aldehydic, pungent	1	Pastry, fatty	4	/	-	Green, fatty	2	/	-	Fatty, pungent	2
12	*p*-Cymene	/	-	/	-	Fragrant, fresh	4	Fragrant and sweet	2	Rice-like	3	/	-
13	2,2,6-Trimethyl-cyclohexanone	Rice crust	1	Spice, sweet	4	Fragrant, honey	3	/	-	/	-	Cooked rice	3
14	3-Octen-2-one	Herbal	2	/	-	Fruity, floral	3	Fruity	3	Fresh, fruity	3	Herbal	3
15	Benzeneacetaldehyde	Green, nutty	2	Floral, rose	4	Rose-like	3	Green, rose	3	Green, floral	3	/	-
16	*trans*-β-Ocimene	/	-	Herbal	4	Floral	4	/	-	Floral, herbal	2	Floral, herbal	3
17	(*E*)-2-octenal	Green, grassy	2	/	-	Fresh, nutty	3	Green, fresh	2	Fresh	3	Nutty	3
18	Acetophenone	Caramel	3	Fresh, floral	4	/	-	Sweet, floral	3	Sweet	3	Sweet, floral	3
19	2-Acetylpyrrole	/	-	Caramel	4	Caramel, coffee	2	Sweet	3	Coffee, sweet	3	Caramel	4
20	*trans*-Linalool oxide (furanoid)	Sweet	3	Floral, sweet	3	Floral, sweet	3	Sweet	3	Floral, sweet	3	Floral, sweet	4
21	3-Ethyl-2,5-dimethylpyrazine	Roasted	2	Coffee, bitter	4	Coffee	3	Roasted, nutty	2	Coffee	3	Coffee	3
22	2,2'-Methylenebis-furan	/	-	Roasted, bitter	4	Coffee	3	Roasted	2	Coffee	3	Coffee, bitter	3
23	3,5-Octadien-2-one	/	-	/	-	Mushroom, herbal	3	Fatty, nutty	2	Earthy, herbal	3	Mushroom, sweet	3
24	Methyl benzoate	/	-	/	-	Leather	2	Floral, fresh	3	Floral, leather	3	/	-
25	Linalool	Floral	3	Floral, citrus	4	Floral, sweet	3	/	-	Floral, fresh	3	Floral	3
26	Nonanal	Floral, fresh	3	Floral, sweet	3	Floral, fruity	3	Floral, caramel	3	Floral, fresh	4	Floral	4

27	6-Methyl-3,5-heptadiene-2-one	Sweet	2	/	-	Coconut, sweet	2	/	-	Coconut creamy	3	/	-
28	Phenylethyl alcohol	/	-	/	-	Floral, sweet	3	Floral, fruity	2	Sweet	4	Sweet	3
29	Methyl octanoate	Green, fresh	3	/	-	Fruity, green	3	Fruity, sweet	3	/	-	/	-
30	*trans*-Alloocimene	/	-	Green	4	Green, fresh	3	Fresh, floral	3	Green, grassy	3	Fresh, floral	4
31	5-Ethyl-6-methyl-3E-hepten-2-one	/	-	Cucumber, fresh	3	Green, grassy	1	Green	3	/	-	Green, fresh	3
32	(*E*,*Z*)-2,6-Nonadienal	Cucumber, fresh	3	Fresh	4	Green apple	3	Green	3	Cucumber, fresh	4	Green, grassy	3
33	2-Methyl-3,5-diethylpyrazine	/	-	/	-	/	-	/	-	/	-	/	-
34	(*E*)-2-nonenal	Green	2	/	-	Green apple	3	Green	1	Cucumber, fresh	4	/	-
35	Benzyl acetate	Green	3	Green, fresh	4	Green	3	Fresh, fruity	1	Fresh, plastic	3	/	-
36	Propiophenone	Hay	3	Green, fresh	4	Green	3	Green, fresh	1	Fresh	3	/	-
37	Naphthalene	Peppery	3	/	-	Pungent, earthy	4	/	-	Earthy	3	Pungent	2
38	1-Furfurylpyrrole	Green, pungent	2	Roasted	3	Pungent, plastic	3	Roasted	1	Green, pesticide	3	/	1
39	Methyl salicylate	/	-	Minty, fresh	4	Herbal	1	Minty, herbal	2	Minty, wintergreen	3	Wintergreen, herbal	2
40	Safranal	/	-	/	-	Herbal	2	Metallic	1	Herbal, rusty	2	/	-
41	(*E*,*E*)-2,4-Nonadienal	Fruity	2	/	-	Green, grassy	3	Fruity, fresh	3	Green	3	Green, fruity	3
42	Phenethyl acetate	/	-	Floral, sweet	3	Floral	4	Floral, sweet	2	Floral, fresh	3	Cucumber	3
43	(*E*)-2-decenal	Green	2	/	-	Green, fresh	4	Fruity, fresh	2	Green	2	/	-
44	α-citral	Citrus	2	/	-	Fresh, lemon	2	/	-	Citrus, fresh	3	/	-
45	Indole	/	-	Floral, fresh	2	/	-	/	-	Floral	2	Leather	2
46	γ-Nonanolactone	/	-	Creamy, floral	3	Coconut creamy	2	/	-	Sweet	2	Floral, sweet	3
47	Hexyl hexanoate	Fruity, floral	2	/	-	/	-	Floral, honey	4	/	-	Fruity, floral	3
48	(*E*)-2-Hexenyl hexanoate	Citrus, fruity	2	/	-	Fruity, sweet	3	/	-	Floral, sweet	4	Floral	3
49	α-Cedrene	Sweet	2	Floral, fresh	3	Floral, sweet	3	/	-	Sweet, fruity	2	Fruity, woody	2
50	α-Ionone	Floral	2	/	-	Sweet, floral	1	/	-	Sweet, floral	2	Sweet	1
51	Coumarin	Floral	4	Floral	3	Milk-like, sweet	4	Floral, fruity	2	Floral	2	Floral, milk-like	3
52	Phenethyl butyrate	Floral, sweet	3	Floral	4	Floral, fruity	4	Floral, fruity	2	Floral, sweet	2	Floral, sweet	3
53	(*E*)-β-Famesene	/	-	Floral, sweet	1	Sweet	3	/	-	Floral	2	/	-
54	*trans*-β-Ionone	Rose, floral	3	/	-	Rose, milk	3	Coconut creamy	3	Coconut creamy	3	Floral, creamy	3
55	Calamenene	Fresh, herbal	2	Fresh and cool	2	/	-	/	-	/	-	Fresh and cool	2
56	Dihydroactinidiolide	Herbal, fresh	3	/	-	Herbal	3	/	-	Clean, essential oil	3	/	-
